# Aberrant Transcription-Factor Expression and Regulatory microRNAs in Metastatic Breast Cancer

**DOI:** 10.3390/cells15191815

**Published:** 2026-10-05

**Authors:** Eman Hillal Althubaiti

**Affiliations:** Department of Biotechnology, College of Science, Taif University, Taif 21944, Saudi Arabia; i.althubaiti@tu.edu.sa

**Keywords:** breast cancer, metastasis, transcription factors, microRNAs, epithelial–mesenchymal plasticity, miR-200c, FOXP3, ZEB1, circulating biomarkers

## Abstract

Metastatic breast cancer is driven by reversible cell-state programs shaped by transcription factors (TFs) and microRNAs (miRNAs). Dysregulated TFs integrate oncogenic signaling, hypoxia, inflammation, lineage identity, mechanical cues, and chromatin state, whereas miRNAs buffer or reinforce these programs through post-transcriptional repression. Their reciprocal interactions form dynamic regulatory circuits controlling epithelial–mesenchymal plasticity, stem-like behavior, invasion, dissemination, dormancy, organ-specific colonization, immune escape, and treatment resistance. This narrative review examines major TF-miRNA modules implicated in breast cancer metastasis, including ZEB1/2-miR-200/miR-205, TWIST1-miR-10b, MYC-miR-9, FRA-1-miR-34, FOXM1-miR-671-5p, HIF-1α-miR-18a, mutant p53/HIF-1α-miR-30d, STAT3-miR-21/miR-34a, and the context-dependent FOXP3-miR-200c/miR-141 network. Evidence from experimental models, paired primary-metastatic tissues, circulating tumor cells, extracellular vesicles, and plasma cohorts is integrated with emphasis on disease stage, molecular subtype, and biological compartment. Tissue and circulating miRNA signals are not interchangeable, and FOXP3-miR-200c relationships appear compartment- and model-dependent. Clinical translation remains limited by tumor heterogeneity, pre-analytical variability, incomplete cellular resolution, and limited causal validation. Prospective, subtype-stratified studies integrating spatial, single-cell, metastatic-tissue, and standardized liquid-biopsy analyses are required before TF-miRNA signatures can guide clinical management.

## 1. Introduction

Breast cancer remains the most frequently diagnosed malignancy in women worldwide, with approximately 2.3 million new cases and 670,000 deaths estimated in 2022 [1,2,3]. Mortality is driven predominantly by distant dissemination rather than by the primary tumor [4]. Metastasis is a multistep evolutionary and ecological process encompassing local invasion, intravasation, survival as single circulating tumor cells (CTCs) or clusters, extravasation, dormancy, and eventual colonization of permissive organs [5,6,7].

Transcription factors translate extracellular signals and chromatin context into coordinated gene-expression programs [8]. In breast cancer, recurrently activated TFs include ZEB1/2, SNAI1/2, TWIST1, FOXM1, MYC, STAT3, NF-κB, HIF-1α, AP-1/FRA-1, and YAP/TAZ-TEAD complexes, whereas lineage-maintaining or tumor-restraining factors such as GATA3 can be lost [9,10,11]. miRNAs, typically 19–24 nucleotides long, bind partially complementary sequences in target transcripts and reduce mRNA stability or translation [12]. Because one miRNA can regulate many targets and one transcript can be regulated by multiple miRNAs, TF-miRNA interactions form networks rather than isolated one-to-one axes [13,14].

Aberrant TF expression can arise from gene amplification, mutation, altered enhancer activity, promoter methylation, changes in protein stability, or persistent upstream signaling [15,16]. These mechanisms are clinically important because an unchanged TF mRNA level does not exclude high transcriptional activity [17]. HIF-1α is principally stabilized post-translationally under hypoxia; STAT3 and NF-κB depend on phosphorylation and nuclear localization. YAP/TAZ are also controlled by mechanical and Hippo-pathway signals [18,19]. Conversely, high FOXM1 transcript abundance may partly reflect proliferation [20,21]. A rigorous TF-miRNA study should therefore distinguish transcript abundance, protein abundance, subcellular localization, DNA-binding activity, and downstream target-gene output.

The classical epithelial–mesenchymal transition (EMT) model is useful but incomplete. EMT can generate cells with stem-like properties [22,23,24], but tumor cells frequently occupy partial or hybrid epithelial/mesenchymal states, and transitions between these states can be more important than a terminal mesenchymal phenotype [25,26,27]. Lineage-tracing experiments showed that a canonical EMT program was not strictly required for lung metastasis in some breast cancer models, although it contributed to chemoresistance [28]. CTC clusters, which retain cell–cell contacts and can display hybrid phenotypes, are highly metastatic oligoclonal precursors [29]. Consequently, this review uses the term epithelial–mesenchymal plasticity (EMP) and evaluates TF-miRNA relationships in relation to the specific stage of metastasis, tissue compartment, and breast cancer subtype.

## 2. Scope and Literature Approach

A targeted narrative literature search of PubMed was conducted from database inception through 1 June 2026. The search strategy and evidence-selection process were designed to improve transparency and reproducibility in accordance with general recommendations for conducting and reporting narrative reviews [30,31]. The core Boolean search combined terms related to breast cancer, miRNAs, transcription factors, and metastatic progression as follows: (“breast cancer” OR “breast neoplasm” OR “metastatic breast cancer”) AND (microRNA OR miRNA*) AND (“transcription factor*” OR ZEB1 OR ZEB2 OR SNAIL OR SNAI1 OR SNAI2 OR TWIST1 OR FOXM1 OR STAT3 OR HIF1A OR MYC OR GATA3 OR FOXP3) AND (metastas* OR invasion OR “epithelial-mesenchymal transition” OR “epithelial-mesenchymal plasticity” OR “circulating tumor cell*” OR “extracellular vesicle*” OR exosome* OR “liquid biopsy” OR “therapy resistance”). Additional targeted searches combined individual TFs or miRNAs with the terms lncRNA, circRNA, ceRNA, premetastatic niche, brain metastasis, bone metastasis, lung metastasis, liver metastasis, and treatment resistance. Peer-reviewed original studies were prioritized when they included direct genetic or pharmacological perturbation, miRNA-target validation, reporter or rescue experiments, and/or in vivo metastasis assays. Particular consideration was also given to studies using paired primary-metastatic human specimens and clinical cohorts reporting survival, disease progression, recurrence, or treatment-response outcomes. Relevant review articles were used to contextualize emerging mechanisms and identify additional primary studies, but mechanistic conclusions were based predominantly on original research. Non-peer-reviewed preprints, conference abstracts, studies unrelated to breast cancer, and purely computational miRNA-target predictions lacking experimental validation were not used as primary evidence for mechanistic conclusions. Consistent with the narrative-review design, study selection was qualitative rather than systematic; quantitative meta-analysis was not performed, and omission of a study should not be interpreted as evidence of absence [30,31].

## 3. Biological Architecture of TF-miRNA Networks

### 3.1. Sources of Transcription-Factor Dysregulation

TF dysregulation in metastatic breast cancer reflects the combined effects of genomic alterations, chromatin remodeling, oncogenic signaling, and microenvironmental selection [32]. These processes influence TF abundance and activity by modifying chromatin accessibility, cofactor recruitment, and signal-dependent activation. Consistent with the broader role of epigenetic regulation in cancer, DNMT-targeting studies in NSCLC and colorectal cancer models have shown that demethylation can restore tumor-suppressor expression while altering regulatory miRNA profiles, further illustrating the interaction between DNA methylation and post-transcriptional regulatory networks [33,34]. Consequently, expression alone may not accurately represent functional TF output [35]. Organ-specific stromal and cytokine signals can further establish transcriptional states that differ between primary tumors and metastatic lesions [36]. Therapy imposes additional selective pressure by enriching drug-tolerant states and promoting clonal evolution [37]. Longitudinal profiling of untreated primary tumors, residual disease, and progressing metastases is therefore needed to define clinically relevant changes in TF activity.

### 3.2. Beyond Pairwise TF-miRNA Loops: ceRNA Networks

TF-miRNA circuits operate within a broader post-transcriptional regulatory environment that includes competing endogenous RNA (ceRNA) networks. Long non-coding RNAs (lncRNAs), circular RNAs (circRNAs), and selected mRNAs can share miRNA-response elements and thereby influence the availability and downstream activity of regulatory miRNAs [38,39]. Accordingly, TF-miRNA relationships should not always be interpreted as isolated one-to-one interactions, because competing RNA species can modify the strength and functional output of these networks and contribute to epithelial–mesenchymal plasticity, metastatic progression, and treatment resistance [40].

Several breast cancer models illustrate this additional regulatory layer. The lncRNA H19 modulates EMT/MET plasticity during metastatic progression through differential interactions with miR-200b/c and let-7b [41]. Similarly, circANKS1B promotes breast cancer invasion and metastasis by sequestering miR-148a-3p and miR-152-3p, thereby increasing USF1 expression and activating TGF-β1/Smad signaling [42]. A TF-linked example is ZEB1-induced circWWC3, which functions as a competing RNA for miR-26b-3p and miR-660-3p and promotes Ras-pathway activation and metastatic progression [43].

Collectively, these findings indicate that apparently simple TF-miRNA switches may be embedded within higher-order ceRNA networks that modify miRNA accessibility and downstream regulatory programs.

**Figure 1:**
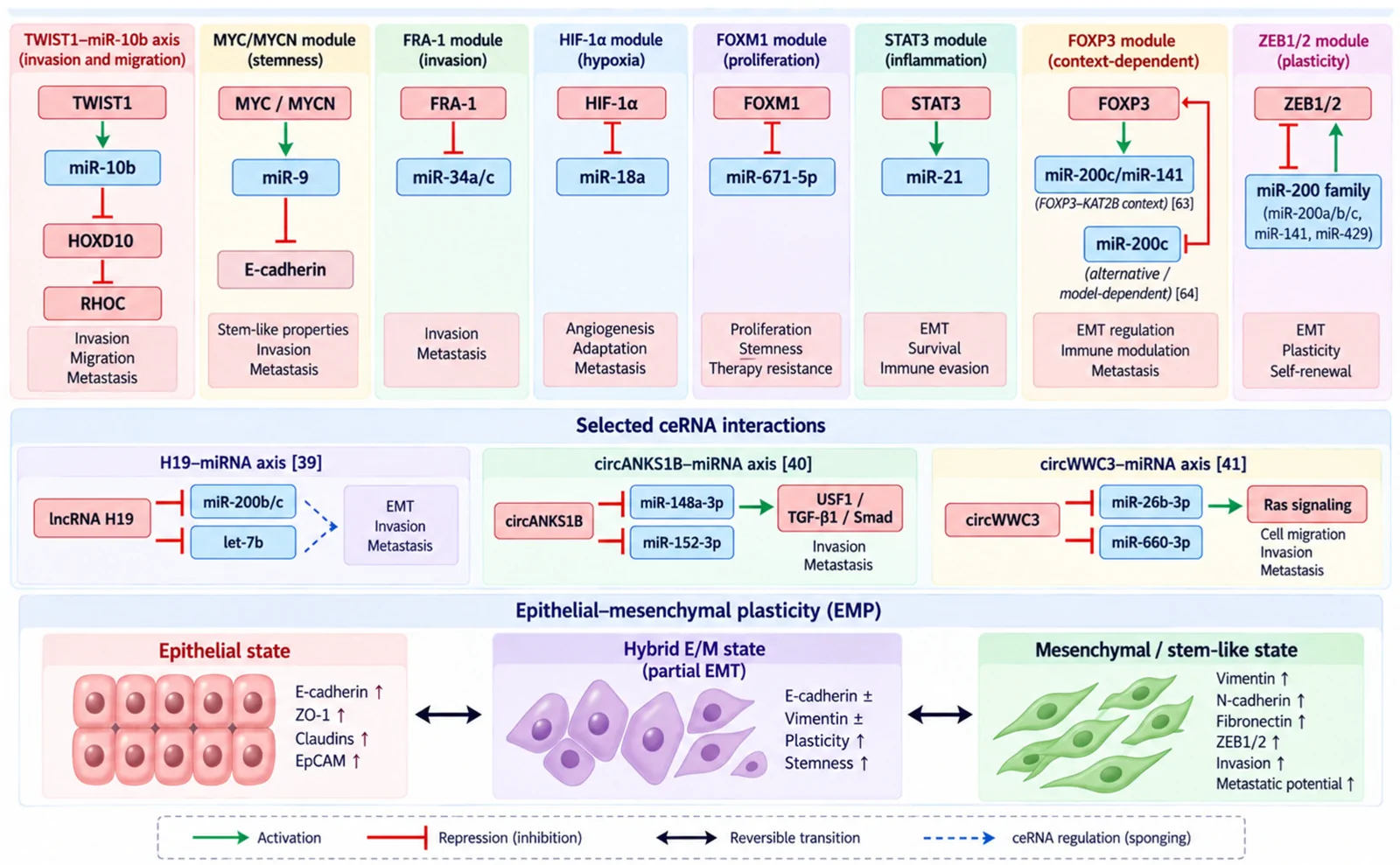
Major transcription factor (TF)–microRNA (miRNA) circuits and selected competing endogenous RNA (ceRNA) interactions regulating epithelial–mesenchymal plasticity in metastatic breast cancer. Highlighted networks include the H19-, circANKS1B-, and circWWC3-mediated ceRNA interactions and the context-dependent FOXP3–miR-200c/miR-141 axis.

Nevertheless, ceRNA activity should not be inferred solely from predicted sequence complementarity. Strong mechanistic claims require evidence of appropriate RNA abundance and subcellular co-localization, direct miRNA binding, perturbation of the candidate ceRNA, and rescue of the downstream phenotype. Incorporating these criteria is particularly important when interpreting ceRNA contributions to bistable or multistable metastatic cell states. The major intracellular TF-miRNA and ceRNA networks contributing to reversible epithelial–mesenchymal plasticity are summarized in Figure 1.

## 4. Major TF-miRNA Circuits in Metastatic Breast Cancer

### 4.1. ZEB1/2-miR-200/miR-205: The Central Plasticity Switch

The most extensively validated circuit links the E-cadherin repressors ZEB1 and ZEB2 with the miR-200 family (miR-200a/b/c, miR-141, and miR-429) and miR-205. Loss of these miRNAs increases ZEB1/2, represses CDH1, and promotes migration; their restoration induces a more epithelial phenotype [44,45]. Conversely, ZEB factors repress miR-200 transcription, generating a reciprocal switch. In breast cancer, a poised ZEB1 promoter enables non-stem basal cells to enter a stem-like state in response to microenvironmental signals, connecting EMP to tumor-initiating capacity [46].

This circuit is stage dependent. In one syngeneic mouse model, miR-200 restoration enhanced lung and liver colonization [47], whereas in xenograft models, miR-200b/200c/429 reduced invasion and metastasis through both ZEB-dependent and moesin-dependent mechanisms [48]. NF-κB activation can converge on ZEB1/2 while suppressing miR-200, and re-expression of miR-200 reduces pulmonary metastasis in this context [49]. The PELP1-HDAC2 complex provides an epigenetic mechanism for miR-200 repression and EMT [50]. Complementary evidence from non-breast carcinoma models indicates that pharmacological DNA demethylation can restore CDH1/E-cadherin expression, further supporting the reversibility of epigenetically reinforced epithelial-marker silencing [51,52]. Methylation of miR-200b promoters is associated with receptor status and was increased in metastatic lymph nodes relative to matched primary tumors [53]. Thus, low miR-200 in an invasive front and high miR-200 in a proliferative metastatic lesion are not necessarily contradictory.

Extracellular vesicle biology further complicates interpretation. miR-200-containing vesicles released by highly metastatic breast cancer cells can transfer metastatic capacity to less aggressive cells [54]. This may partly explain why high circulating miR-200 family levels can accompany progressive metastatic disease even though intracellular miR-200 suppresses EMT in many experimental settings.

### 4.2. TWIST1-miR-10b-HOXD10/RHOC

TWIST1 is a prototypical EMT-inducing transcription factor with a well-established role in breast cancer dissemination. A seminal study by Ma et al. demonstrated that TWIST1 directly induces miR-10b transcription by binding to its regulatory region. Increased miR-10b subsequently suppresses HOXD10, relieving repression of the pro-metastatic GTPase RHOC and thereby promoting tumor-cell invasion and distant metastasis [55]. Clinical evidence has also linked increased circulating miR-10b with advanced and metastatic breast cancer, with higher serum levels reported in metastatic than in non-metastatic disease [56]. This axis therefore represents a TF-driven oncomiR program that translates transcriptional dysregulation into pro-invasive and metastatic phenotypes. Nevertheless, despite renewed interest in miR-10b as a potential biomarker and therapeutic target, current evidence remains insufficient to support its use as a stand-alone clinical marker, and larger prospective studies with standardized sampling and analytical procedures are still required [57].

### 4.3. MYC-miR-9-CDH1 and SNAIL/FRA-1-miR-34

MYC and MYCN activate miR-9, which directly targets CDH1, increases β-catenin signaling, and promotes angiogenesis and metastasis [58]. This provides a direct route from oncogenic transcription to loss of epithelial adhesion. In parallel, the p53-responsive miR-34 family restrains invasion by targeting the AP-1 component FRA-1; miR-34a/c levels are reduced in metastatic breast cancer cells and tumors, whereas restoration decreases invasion and metastasis [59]. miR-34 also intersects with SNAIL-centered networks and stemness, although some detailed feed-forward mechanisms were established in colorectal rather than breast models and should not be transferred uncritically.

Adipocyte-derived leptin can activate STAT3 and PAI-1 while suppressing miR-34a, thereby linking obesity-associated stromal signaling to EMT and metastasis [60]. This is clinically important because the metastatic phenotype may reflect both cancer-cell genotype and systemic metabolic context.

### 4.4. FOXM1-miR-671-5p and Proliferative Plasticity

FOXM1 is overexpressed across breast cancer subtypes and promotes cell-cycle progression, DNA repair, stemness, invasion, and resistance [61]. miR-671-5p directly restrains FOXM1-associated EMT and DNA-repair programs; progressive loss of miR-671-5p accompanies oncogenic transformation and radio-chemoresistance [62]. The axis is attractive because it couples metastatic plasticity to therapeutic resistance, but most evidence remains preclinical. FOXM1 abundance should also be interpreted with proliferation indices because high expression may partly reflect cell-cycle composition rather than a metastasis-specific program.

### 4.5. Hypoxia-Responsive Networks: HIF-1α-miR-18a and Mutant p53/HIF-1α-miR-30d

Hypoxia is selected for invasive, metabolically adaptable cells and activates HIF-1α. In basal-like breast cancer, miR-18a directly reduces HIF-1α activity and suppresses lung metastasis; low miR-18a therefore removes a brake on the hypoxic metastatic program [63]. A distinct pathway operates in mutant-TP53 breast cancer: mutant p53 cooperates with HIF-1α to induce miR-30d, which represses DGKZ and VPS26B, reorganizes the Golgi apparatus, and promotes release of a pro-metastatic secretome that remodels extracellular matrix and stromal behavior [64]. These findings broaden the TF–miRNA concept beyond cell-autonomous EMT to paracrine preparation of the metastatic niche.

### 4.6. STAT3, NF-κB, and Inflammatory oncomiRNAs

Inflammatory cytokines converge on STAT3 and NF-κB, which induce EMT TFs, survival genes, immune-modulatory factors, and miRNAs. Leukemia inhibitory factor promotes breast cancer EMT and migration through STAT3-dependent miR-21 induction [65]. miR-21 can simultaneously inhibit tumor suppressor pathways, making it a network amplifier rather than a specific readout of one TF. NF-κB also cooperates with ZEB1/2 and miR-200 repression [49]. Because inflammatory miRNAs are expressed by leukocytes and platelets as well as tumor cells, blood-based measurements require rigorous control for cellular contamination and systemic inflammation.

### 4.7. FOXP3-miR-200c/miR-141: A Context-Dependent and Compartment-Sensitive Network

FOXP3 requires especially careful interpretation. In tumor-infiltrating regulatory T cells, FOXP3 marks an immunosuppressive immune population; in breast epithelial tumor cells, FOXP3 can have distinct transcriptional functions and may act as either a tumor suppressor or a context-dependent promoter of dissemination. Tumor-cell FOXP3 has been reported to regulate CXCR4 and responsiveness to CXCL12, directly linking it to chemotactic metastasis [66]. A FOXP3-KAT2B transcriptional axis was shown to regulate miR-200c and miR-141; loss of epithelial FOXP3 reduced these miRNAs, and low expression was associated with metastatic behavior [67].

A 2026 study in metastatic breast cancer models reported the apparently opposite direction: miR-200c restoration reduced FOXP3 expression, migration, invasion, and metastatic spread [68]. This discrepancy may reflect different cellular compartments, models, baseline states, or reciprocal feedback. Technical and analytical differences may contribute to the apparently opposing observations regarding FOXP3 in breast cancer. FOXP3 immunohistochemistry is sensitive to methodological variables, including antibody clone, tissue fixation, antigen-retrieval conditions, staining thresholds, and the distinction between nuclear staining in carcinoma cells and infiltrating immune cells. Importantly, different monoclonal FOXP3 antibodies have produced discordant staining patterns in formalin-fixed, paraffin-embedded tissues, emphasizing the need for antibody validation and, where possible, orthogonal confirmation of tumor-cell FOXP3 expression [69].

FOXP3 is strongly expressed by tumor-infiltrating regulatory T cells (Tregs), whereas FOXP3 expression has also been reported in breast carcinoma cells. Studies evaluating these compartments separately have shown that tumor-cell FOXP3 and FOXP3-positive tumor-infiltrating lymphocytes may have distinct clinicopathological and prognostic associations [70]. Consequently, bulk-tumor RNA or protein measurements may combine carcinoma-cell and immune-cell FOXP3 signals, particularly in immune-rich specimens. Multiplex immunostaining with epithelial and immune markers, laser-capture microdissection, spatial profiling, or single-cell approaches may therefore provide greater cell-of-origin resolution when carcinoma-cell FOXP3 is the specific biological question.

Epigenetic background may provide a further source of model dependence. In breast cancer specimens, FOXP3 promoter methylation has been associated with reduced FOXP3 mRNA and protein expression, supporting an epigenetic contribution to variation in FOXP3 abundance [71]. In parallel, methylation of the miR-200c/miR-141 locus varies among breast cancer models and is associated with invasive phenotype, lymph node metastasis, and ZEB1 activity. ZEB1 depletion has also been shown to reduce methylation of this locus while increasing miR-200c expression, further linking epigenetic regulation to epithelial–mesenchymal plasticity [72]. Thus, differences in baseline methylation and transcriptional state may influence the magnitude—and potentially the apparent direction—of the FOXP3-miR-200c relationship.

Accordingly, the opposing directions reported for the FOXP3-miR-200c relationship should not yet be regarded as universally interchangeable mechanisms. Resolving this discrepancy will require studies that establish cellular origin, use validated FOXP3 detection methods, quantify Treg infiltration, characterize relevant epigenetic states, and perform reciprocal perturbation and rescue experiments within matched breast cancer models.

### 4.8. Lineage and Organotropism Modules

GATA3 maintains luminal differentiation and can reverse EMT in invasive breast cancer cells; its restoration suppresses metastasis [73]. Loss of GATA3 therefore removes a lineage-stabilizing constraint, particularly in dedifferentiated or therapy-resistant disease. Foundational work by Tavazoie et al. identified a group of endogenous miRNAs whose expression is selectively lost as human breast cancer cells acquire metastatic competence. Restoration of miR-335 and miR-126 suppressed metastatic progression in vivo, while miR-206 was also implicated in regulating tumor-cell migration and morphology. Mechanistically, miR-335 inhibited migration and metastasis partly by directly targeting the transcription factor SOX4 and the extracellular matrix component tenascin C (TNC). Loss of miR-335 or miR-126 expression in primary breast tumors was further associated with poor distant metastasis-free survival, establishing these miRNAs as endogenous suppressors of breast cancer metastasis [74].

Additional miRNAs regulate distinct components of metastatic progression and organ-associated dissemination. miR-1258 suppresses breast cancer brain metastasis by directly targeting heparanase (HPSE), thereby reducing tumor-cell invasion and experimental brain colonization [75]. Similarly, miR-101 inhibits breast cancer proliferation, invasion, tumor growth, and lung metastasis by directly targeting CXCR7, with reduced miR-101 expression associated with advanced disease and lymph node metastasis [76]. More recently, miR-338-5p was shown to suppress triple-negative breast cancer proliferation, migration, invasion, and metastatic progression through regulation of the ETS1/NOTCH1 axis [77]. Collectively, these findings demonstrate that metastasis-suppressive miRNAs can regulate multiple stages of breast cancer progression beyond EMT, including tumor-cell invasion, extracellular matrix remodeling, metastatic niche interactions, and organ colonization, as summarized in Table 1.

### 4.9. Extracellular-Vesicle-Mediated miRNA Communication and Premetastatic-Niche Remodeling

TF-miRNA regulation in metastatic breast cancer extends beyond tumor-cell-intrinsic signaling to extracellular-vesicle-mediated intercellular communication. Cancer cells can selectively package miRNAs into extracellular vesicles (EVs), enabling their transfer to endothelial, stromal, and other recipient cells at distant sites. This mechanism provides a functional link between tumor-cell miRNA dysregulation, premetastatic-niche remodeling, vascular permeability, and metastatic colonization. Several mechanistic studies illustrate this process. Breast-cancer-derived EV-associated miR-105 is transferred to endothelial cells, where it directly targets the tight-junction protein ZO-1, disrupts endothelial-barrier integrity, and facilitates metastatic dissemination [78].

Similarly, breast-cancer-secreted miR-122 suppresses glucose uptake by cells within premetastatic tissues, thereby increasing nutrient availability for disseminated tumor cells and promoting metastatic colonization [79].

**Figure 2:**
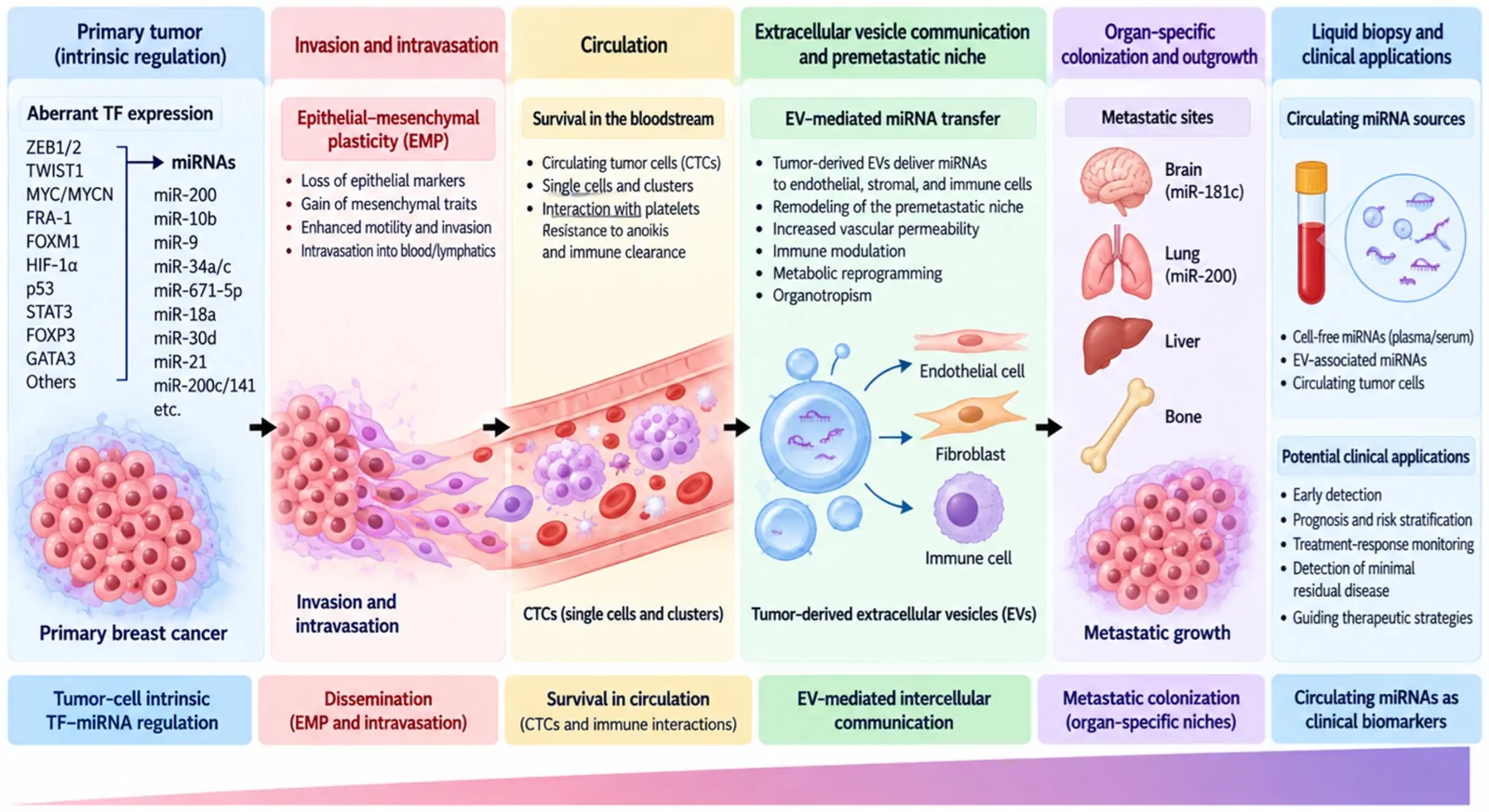
Spatial dynamics of miRNA regulation, extracellular-vesicle communication, and liquid-biopsy compartments during breast cancer metastasis. Tumor-cell-intrinsic miRNA networks regulate invasion and dissemination, whereas EV-associated miRNAs contribute to vascular remodeling, premetastatic-niche formation, and metastatic colonization. Circulating cell-free miRNAs, EV-associated miRNAs, and circulating tumor cells represent distinct liquid-biopsy analytes with potential applications in prognosis, disease monitoring, and treatment-response assessment.

In brain-tropic breast cancer models, EV-associated miR-181c disrupts the blood–brain barrier by targeting PDPK1 and altering cofilin-dependent actin dynamics, thereby facilitating brain metastasis [80]. Together with the previously described transfer of miR-200-containing EVs [54], these findings demonstrate that miRNAs can promote metastatic progression through both cell-autonomous regulatory circuits and intercellular communication. Importantly, biologically active EV-associated miRNAs should be distinguished from circulating miRNAs measured solely as liquid-biopsy biomarkers. The spatial and temporal relationships among tumor-cell-intrinsic miRNA regulation, EV-mediated communication, metastatic colonization, and circulating-miRNA biomarkers are summarized in Figure 2.

## 5. Molecular Subtype and Metastatic-Site Context

### 5.1. Triple-Negative and Basal-like Breast Cancer

TNBC frequently exhibits high ZEB1, TWIST1, SNAIL, FOXC1, FOXM1, HIF-1α, NF-κB, STAT3, and YAP/TAZ activity together with reduced epithelial miRNAs. Basal-like models support miR-18a-HIF-1α [63], miR-671-5p-FOXM1 [62], and miR-338-5p-ETS1/NOTCH1 [77] circuits. However, TNBC is transcriptionally heterogeneous; mesenchymal, immunomodulatory, and luminal androgen receptor states may use different TF–miRNA programs. A universal TNBC miRNA signature is therefore unlikely without molecular stratification.

### 5.2. Hormone-Receptor-Positive Disease

ER-positive tumors retain lineage TFs such as GATA3 and FOXA1, but metastatic progression and endocrine resistance can involve reprogramming of enhancer use, activation of growth-factor TFs, and acquisition of EMP. miR-200 promoter methylation is associated with hormone-receptor status [53]. Loss of lineage identity may be more informative than absolute expression of a single EMT TF. Importantly, an ER-positive metastatic lesion may differ substantially from its primary tumor after years of endocrine selection.

### 5.3. HER2-Positive Disease

HER2 signaling engages AP-1, STAT3, NF-κB, FOXM1, and YAP/TAZ-associated transcriptional programs. Circulating miR-200c/FOXP3 relationships have been explored as predictors of trastuzumab response [81], but the evidence remains hypothesis-generating. A clinically useful signature must add predictive information beyond HER2 expression, disease burden, prior therapy, and established clinical variables.

### 5.4. Organ-Specific Metastasis

Brain, bone, liver, and lung metastases impose different selective pressures. miR-1258-heparanase is particularly relevant to brain metastasis [75], whereas miR-18a-HIF-1α and several miR-200 studies emphasize lung colonization [63]. Organotropism claims should therefore be supported by site-specific models and tissues rather than inferred from generic migration assays.

## 6. Human Correlation and Liquid-Biopsy Evidence

From a translational perspective, three analytically and biologically distinct miRNA compartments should be distinguished: (i) tissue-associated miRNAs, (ii) total circulating or cell-free miRNAs measured in plasma or serum, and (iii) EV-associated miRNAs isolated from circulating vesicle fractions. Tissue-associated miRNAs primarily reflect the cellular composition and regulatory state of the sampled lesion, whereas circulating miRNAs represent a heterogeneous pool derived from tumor cells, blood cells, platelets, tissue injury, protein-bound miRNA complexes, and extracellular vesicles [82]. Importantly, a substantial fraction of circulating miRNAs can be associated with protein complexes such as Argonaute rather than with vesicles, indicating that EV-associated miRNAs represent only a subset of the total circulating miRNA pool [83]. EV-associated measurements are additionally influenced by the methods used for vesicle separation, purification, and characterization [84]. These compartments should therefore not be interpreted interchangeably.

Pre-analytical and analytical factors further complicate comparison across liquid-biopsy studies. Serum and plasma should not be considered interchangeable because coagulation and platelet activation can substantially alter extracellular miRNA profiles, and platform-dependent differences may further affect measured abundance [85]. Hemolysis, residual platelet contamination, blood-processing time, centrifugation protocol, storage conditions, freeze–thaw handling, RNA-extraction efficiency, normalization strategy, and assay platform can all influence circulating-miRNA measurements [86].

For EV-associated miRNAs, additional variability arises from differences in EV separation and characterization methods, which may recover distinct vesicle populations together with variable amounts of non-vesicular extracellular material. Standardized pre-analytical workflows, rigorous sample-quality control, reproducible normalization procedures, and adherence to contemporary EV-characterization guidelines are therefore essential before circulating or EV-associated miRNA signatures can be translated into clinical practice [84].

Paired specimens provide stronger evidence than comparisons of unrelated primary and metastatic cohorts. In matched primary tumors and lymph node metastases, miR-200 family members, miR-205, miR-214, and other miRNAs show site-associated changes [87]. Nevertheless, lymph node involvement is not equivalent to hematogenous distant metastasis, and nodal tissue contains abundant immune cells that influence miRNA profiles. Spatial or single-cell methods are needed to determine whether the observed change arises from carcinoma cells, lymphocytes, stroma, or altered cellular proportions.

Circulating miRNAs are appealing because they are stable and can be measured repeatedly [88]. High circulating miR-200c, alone or with low miR-141, has been associated with unfavorable survival [89]. Broader panels have correlated with CTC burden, progression-free survival, and overall survival in metastatic cohorts [90]. Longitudinal measurement may be more informative than a single baseline determination because serial changes can reflect evolving disease burden, treatment response, and progression while partially reducing inter-individual baseline variability. In patients with metastatic breast cancer, circulating miR-200a, miR-200b, and miR-141 levels decreased after the first completed cycle of a new systemic treatment, whereas all measured miR-200 family members were significantly increased at radiologically confirmed disease progression compared with the post-treatment time point [91]. Nevertheless, prospective validation using standardized sampling, normalization procedures, predefined thresholds, and clinically relevant endpoints remains necessary before routine clinical implementation.

Single-CTC methylation analysis of miR-200 clusters and CDH1 demonstrates the feasibility of measuring EMP-related epigenetic states in rare cells [92]. Integrating CTC phenotype with plasma miRNAs may be more informative than either alone, but technical recovery bias can preferentially capture epithelial or mesenchymal populations, as shown in Table 2.

## 7. Biomarker and Therapeutic Implications

### 7.1. Biomarker Design

A clinically useful TF-miRNA biomarker should be defined according to its biological compartment, breast cancer subtype, metastatic site, treatment setting, and intended clinical use, because diagnostic, prognostic, predictive, and pharmacodynamic biomarkers require different validation frameworks [93]. Liquid-biopsy applications include early detection, prognostic stratification, recurrence or metastasis surveillance, and assessment of therapeutic response; however, the maturity and quality of evidence differ substantially among these applications [94]. Recent breast cancer literature supports particular interest in longitudinal monitoring, because repeated minimally invasive sampling can capture temporal changes in tumor burden, treatment response, and disease progression [95].

In metastatic breast cancer, serial assessment of circulating miR-200 family members together with circulating tumor cells (CTCs) has demonstrated treatment- and progression-associated changes, supporting the potential value of integrated dynamic monitoring [91]. Multiplex signatures are likely to outperform single analytes. A rational panel might include an EMP component (miR-200c/miR-205 with ZEB1), a hypoxia component (miR-18a/HIF-1α), an inflammatory component (miR-21/STAT3), a proliferation-resistance component (miR-671-5p/FOXM1), and a tumor-immune component that distinguishes carcinoma-cell FOXP3 from FOXP3-positive lymphocytes. Such a panel should be tested against, not merely alongside, established clinical predictors.

### 7.2. Therapeutic Targeting

Therapeutic strategies include miRNA mimics to restore metastasis suppressors, antisense oligonucleotides to inhibit oncomiRs, small molecules or degraders that inhibit TFs, and epigenetic agents that reactivate silenced miRNA loci. Delivery remains the dominant challenge: systemic oligonucleotides must reach disseminated tumor cells and relevant organs without provoking immune toxicity or altering beneficial miRNA functions in normal tissues. The stage-specific behavior of miR-200 warns that constitutive restoration could suppress invasion yet favor colonization in some contexts.

Despite strong preclinical rationale, the clinical translation of miRNA therapeutics remains challenging because individual miRNAs regulate multiple transcripts and signaling networks, increasing the risk of sequence-dependent and network-level off-target effects. Systemically administered miRNA mimics and inhibitors also face enzymatic degradation, rapid clearance, nonspecific biodistribution, hepatic and reticuloendothelial uptake, limited tumor penetration, and inefficient endosomal escape [96]. Although lipid- and nanoparticle-based delivery systems can improve RNA stability and cellular uptake, many formulations preferentially accumulate in the liver and may introduce carrier-related toxicity, thereby limiting efficient delivery to extrahepatic metastatic sites [97].

Innate immune activation represents an additional barrier. RNA oligonucleotides and their delivery systems can stimulate endosomal nucleic acid sensors, including TLR7 and TLR8, and provoke inflammatory responses. The clinical experience with the liposomal miR-34a mimic MRX34 illustrates this limitation: although pharmacodynamic target modulation and preliminary antitumor activity were observed, the phase I trial was terminated early after serious immune-mediated adverse events, including four treatment-associated deaths [98]. These findings emphasize that successful miRNA therapy will require tumor-selective delivery, controlled pharmacokinetic exposure, minimization of innate immune activation, rigorous off-target assessment, and direct confirmation of target engagement in tumor tissue.

MiRNA dysregulation can also contribute to therapeutic resistance and the persistence of stem-like tumor-cell states. In luminal breast cancer models, loss of miR-27b increases expression of ENPP1, which promotes an ABCG2-associated side population with cancer-stem-cell properties and drug resistance. Restoration of miR-27b or suppression of ENPP1 reduces this phenotype, whereas ENPP1 upregulation reinforces stem-like characteristics and resistance to docetaxel [99].

Direct TF inhibition is also difficult because many TFs lack classical drug-binding pockets and are required in normal tissues. Network-based approaches may be more feasible. Examples include blocking upstream STAT3 or HIF signaling, interrupting FOXM1 transcriptional dependencies, modulating YAP/TAZ-TEAD activity, or combining miRNA delivery with targeted nanoparticles. Pharmacodynamic biopsies should verify that the intended TF-miRNA circuit, rather than an unrelated stress response, was modified.

## 8. Methodological Limitations and Reasons for Inconsistent Findings

First, correlation does not establish regulatory causality. A statistically significant inverse relationship between miRNA and TF in patient samples may arise from subtype composition or cellular admixture. Direct binding should be demonstrated with reporter assays containing wild-type and mutated miRNA-response elements, followed by rescue experiments and reciprocal mimic/inhibitor perturbation.

Second, migration and invasion assays do not reproduce the full metastatic cascade. Strong claims require orthotopic models, spontaneous metastasis, organ-specific colonization, and ideally immune-competent systems. Tail-vein experiments test survival and colonization more than primary-site invasion.

Third, miRNA measurements are highly sensitive to specimen processing and normalization. Serum versus plasma, anticoagulant, centrifugation, storage, hemolysis, RNA extraction, spike-ins, endogenous controls, and assay platform can change results. U6 is often unsuitable as a universal extracellular normalizer. Reporting should follow established miRNA and liquid-biopsy quality frameworks.

Fourth, breast cancer models are not interchangeable. MDA-MB-231, 4T1, MCF-7, HER2-amplified lines, patient-derived organoids, and metastatic biopsies represent distinct genotypes, species, and microenvironments. Findings should be replicated in more than one model and stratified by receptor status and metastatic site.

## 9. Research Priorities

Future studies should prioritize paired primary tumors, longitudinal blood samples, and biopsies from progressing metastatic sites. Single-cell RNA sequencing alone is insufficient for mature miRNAs; it should be combined with small-RNA sequencing, spatial transcriptomics, multiplexed in situ hybridization, chromatin accessibility, and protein-level TF localization. Lineage tracing and barcoding can determine whether a TF–miRNA state precedes dissemination or emerges after therapy.

Causal inference will improve through CRISPR interference/activation of miRNA loci and TF enhancers, endogenous editing of miRNA-binding sites, and rescue experiments at physiologic expression levels. Patient-derived organoids and explants should include stromal and immune compartments. For FOXP3, studies must separately quantify carcinoma-cell FOXP3, FOXP3-positive regulatory T cells, miR-200c/miR-141, and CXCR4 signaling.

Prospective biomarker trials should pre-register the assay, normalization strategy, endpoints, and cutoffs; include independent validation; and test incremental clinical utility. Response monitoring should be anchored to imaging and standardized progression criteria. Ultimately, a dynamic state score integrating TF activity, miRNA abundance, CTC phenotype, and tumor burden may be more informative than a binary marker.

## 10. Conclusions

Aberrant TF expression and regulatory miRNAs form reciprocal, plastic networks that coordinate metastatic breast cancer rather than acting as isolated biomarkers. ZEB1/2-miR-200 remains the paradigmatic circuit, but TWIST1-miR-10b, MYC-miR-9, FRA-1-miR-34, FOXM1-miR-671-5p, HIF-centered networks, inflammatory STAT3/NF-κB-miRNA programs, and the compartment-dependent FOXP3-miR-200c/miR-141 axis expand the landscape. Their clinical correlations are biologically meaningful but strongly dependent on disease stage, subtype, metastatic site, and specimen source. Translation will require mechanistic validation, spatial and single-cell resolution, standardized liquid-biopsy workflows, and prospective studies designed around specific clinical decisions. The field should move from asking whether one miRNA is simply high or low to determining which cell expresses it, which TF program it regulates, and at what point in the metastatic cascade.

## Figures and Tables

**Table 1:** Representative TF-miRNA modules implicated in breast cancer metastasis.

TF/Module	Regulatory miRNA	Predominant Alteration	Metastatic Consequence	Key Refs.
ZEB1/2	miR-200 family; miR-205	miRNA loss/ZEB gain during invasion; possible miRNA recovery during colonization	EMP, stem-like plasticity, invasion, context-dependent colonization	[44,45,46,47,48]
TWIST1	miR-10b	TWIST1-driven miR-10b induction	HOXD10 repression, RHOC activation, invasion and dissemination	[55]
MYC/MYCN	miR-9	miR-9 induction	Direct CDH1 repression, β-catenin activation, angiogenesis	[58]
FRA-1	miR-34a/c	miR-34 loss	Enhanced EMT, invasion, stemness, and metastatic competence	[59]
FOXM1	miR-671-5p	miR-671-5p loss/FOXM1 gain	EMP, DNA repair, radio-chemoresistance	[62]
HIF-1α	miR-18a	miR-18a loss	Hypoxia adaptation and lung metastasis	[63]
Mutant p53 + HIF-1α	miR-30d	miR-30d induction	Golgi remodeling and a pro-metastatic secretome	[64]
STAT3	miR-21; miR-34a	miR-21 induction and/or miR-34a suppression	Inflammation-linked EMP, migration, and survival	[60,65]
FOXP3	miR-200c; miR-141	Bidirectional, model- and compartment-dependent	CXCR4 signaling, EMP, invasion, treatment-response associations	[66,67,68]
GATA3	Multiple indirect networks	GATA3 loss	Loss of luminal identity and increased metastatic plasticity	[73]
ETS1/NOTCH1	miR-338-5p	miR-338-5p loss	TNBC proliferation, migration, invasion	[77]

**Table 2:** Selected clinical correlations and their interpretation.

Material/Context	Reported Association	Interpretive Value	Main Limitation
Paired primary and lymph-node metastases	Reduced miR-200/miR-205/miR-214 in nodal metastases in selected cohorts [87].	Supports evolution of regulatory states during spread	Nodal tissue is immune-rich and not equivalent to distant metastasis
Primary tumor outcome cohorts	Loss of metastasis-suppressive miRNAs, including miR-335 and miR-126, was associated with unfavorable distant metastasis-free survival [74].	Prognostic enrichment	Retrospective cutoffs and subtype confounding
Plasma/serum in MBC	miR-200 family and multi-miRNA panels associated with CTCs, progression, or survival [89,90].	Repeatable liquid-biopsy signal	Hemolysis, platelets, normalization, and disease burden
Extracellular vesicles	miR-200 transfer can promote metastatic traits [54]	Mechanistic bridge between circulating and tissue findings	Isolation methods and vesicle-source attribution
Single CTCs	Methylation states of miR-200 clusters and CDH1 are measurable [92]	Potential dynamic EMP readout	Low cell numbers and capture bias
Tumor-cell FOXP3 versus FOXP3 + Tregs	Different compartments can show opposing associations [70].	Potential composite tumor-immune biomarker	Routine assays often do not resolve cell of origin

## Data Availability

No new data were created or analyzed in this study. Data sharing is not applicable to this article.

## References

[1] Kim J., Harper A., McCormack V., Sung H., Houssami N., Morgan E., Mutebi M., Garvey G., Soerjomataram I., Fidler-Benaoudia M.M. (2025). Global patterns and trends in breast cancer incidence and mortality across 185 countries. Nat. Med..

[2] Arnold M., Morgan E., Rumgay H., Mafra A., Singh D., Laversanne M., Vignat J., Gralow J.R., Cardoso F., Siesling S. (2022). Current and future burden of breast cancer: Global statistics for 2020 and 2040. Breast.

[3] Gu H., Wang R., Beeraka N.M., Lu P., Zhao X., Li L., Liu Y., Kandula D.R., Fan R., Gayathri T. (2026). Global burden and trends of breast cancer: GLOBOCAN 2022 estimates of incidence and mortality in 185 countries. Chin. Med. J..

[4] Borriello L., Condeelis J., Entenberg D., Oktay M.H. (2021). Breast Cancer Cell Re-Dissemination from Lung Metastases-A Mechanism for Enhancing Metastatic Burden. J. Clin. Med..

[5] Majidpoor J., Mortezaee K. (2021). Steps in metastasis: An updated review. Med. Oncol..

[6] Aceto N. (2020). Bring along your friends: Homotypic and heterotypic circulating tumor cell clustering to accelerate metastasis. Biomed. J..

[7] Herath S., Razavi Bazaz S., Monkman J., Ebrahimi Warkiani M., Richard D., O’Byrne K., Kulasinghe A. (2020). Circulating tumor cell clusters: Insights into tumour dissemination and metastasis. Expert Rev. Mol. Diagn..

[8] Weidemüller P., Kholmatov M., Petsalaki E., Zaugg J.B. (2021). Transcription factors: Bridge between cell signaling and gene regulation. Proteomics.

[9] Feldker N., Ferrazzi F., Schuhwerk H., Widholz S.A., Guenther K., Frisch I., Jakob K., Kleemann J., Riegel D., Bönisch U. (2020). Genome-wide cooperation of EMT transcription factor ZEB1 with YAP and AP-1 in breast cancer. EMBO J..

[10] Zanconato F., Forcato M., Battilana G., Azzolin L., Quaranta E., Bodega B., Rosato A., Bicciato S., Cordenonsi M., Piccolo S. (2015). Genome-wide association between YAP/TAZ/TEAD and AP-1 at enhancers drives oncogenic growth. Nat. Cell Biol..

[11] Thompson B.J. (2020). YAP/TAZ: Drivers of Tumor Growth, Metastasis, and Resistance to Therapy. Bioessays.

[12] Seida M., Ogami K., Yoshino S., Suzuki H.I. (2025). Fine Regulation of MicroRNAs in Gene Regulatory Networks and Pathophysiology. Int. J. Mol. Sci..

[13] Bracken C.P., Khew-Goodall Y., Goodall G.J. (2015). Network-Based Approaches to Understand the Roles of miR-200 and Other microRNAs in Cancer. Cancer Res..

[14] Bracken C.P., Scott H.S., Goodall G.J. (2016). A network-biology perspective of microRNA function and dysfunction in cancer. Nat. Rev. Genet..

[15] Bushweller J.H. (2019). Targeting transcription factors in cancer—From undruggable to reality. Nat. Rev. Cancer.

[16] Libermann T.A., Zerbini L.F. (2006). Targeting transcription factors for cancer gene therapy. Curr. Gene Ther..

[17] Brent M.R. (2016). Past Roadblocks and New Opportunities in Transcription Factor Network Mapping. Trends Genet..

[18] Dasgupta I., McCollum D. (2019). Control of cellular responses to mechanical cues through YAP/TAZ regulation. J. Biol. Chem..

[19] Seo J., Kim J. (2018). Regulation of Hippo signaling by actin remodeling. BMB Rep..

[20] Dong Q., Wang D., Song C., Gong C., Liu Y., Zhou X., Yue J., Hu Y., Liu H., Zhu L. (2024). ABL1-mediated phosphorylation promotes FOXM1-related tumorigenicity by Increasing FOXM1 stability. Cell Death Differ..

[21] Liao G.B., Li X.Z., Zeng S., Liu C., Yang S.M., Yang L., Hu C.J., Bai J.Y. (2018). Regulation of the master regulator FOXM1 in cancer. Cell Commun. Signal..

[22] Wilson M.M., Weinberg R.A., Lees J.A., Guen V.J. (2020). Emerging Mechanisms by which EMT Programs Control Stemness. Trends Cancer.

[23] Brabletz S., Schuhwerk H., Brabletz T., Stemmler M.P. (2021). Dynamic EMT: A multi-tool for tumor progression. EMBO J..

[24] Gooding A.J., Schiemann W.P. (2020). Epithelial-Mesenchymal Transition Programs and Cancer Stem Cell Phenotypes: Mediators of Breast Cancer Therapy Resistance. Mol. Cancer Res..

[25] Jolly M.K., Boareto M., Huang B., Jia D., Lu M., Ben-Jacob E., Onuchic J.N., Levine H. (2015). Implications of the Hybrid Epithelial/Mesenchymal Phenotype in Metastasis. Front. Oncol..

[26] Jolly M.K., Somarelli J.A., Sheth M., Biddle A., Tripathi S.C., Armstrong A.J., Hanash S.M., Bapat S.A., Rangarajan A., Levine H. (2019). Hybrid epithelial/mesenchymal phenotypes promote metastasis and therapy resistance across carcinomas. Pharmacol. Ther..

[27] Subbalakshmi A.R., Ashraf B., Jolly M.K. (2022). Biophysical and biochemical attributes of hybrid epithelial/mesenchymal phenotypes. Phys. Biol..

[28] Fischer K.R., Durrans A., Lee S., Sheng J., Li F., Wong S.T., Choi H., El Rayes T., Ryu S., Troeger J. (2015). Epithelial-to-mesenchymal transition is not required for lung metastasis but contributes to chemoresistance. Nature.

[29] Schuster E., Taftaf R., Reduzzi C., Albert M.K., Romero-Calvo I., Liu H. (2021). Better together: Circulating tumor cell clustering in metastatic cancer. Trends Cancer.

[30] Green B.N., Johnson C.D., Adams A. (2006). Writing narrative literature reviews for peer-reviewed journals: Secrets of the trade. J. Chiropr. Med..

[31] Baethge C., Goldbeck-Wood S., Mertens S. (2019). SANRA-a scale for the quality assessment of narrative review articles. Res. Integr. Peer Rev..

[32] Zhu Q., Tekpli X., Troyanskaya O.G., Kristensen V.N. (2020). Subtype-specific transcriptional regulators in breast tumors subjected to genetic and epigenetic alterations. Bioinformatics.

[33] El Nobey M.A., Shaikh Omar A.M., Batawi A.H., Alharthi A., Althubaiti E.H., Alghamdi M.A., Altalhi S.A., Bakhsh T., Al Aamri Z.M. (2026). Integrated Bioinformatic and Experimental Analysis of PTEN and DNMT1 Regulation in NSCLC. Biomedicines.

[34] El Nobey M.A., Shaikh Omar A.M., Zari A., Alharthi A., Alghamdi M.A., Althubaiti E.H., Al-Shaeri M.A., Bakhsh T., El Hamidy S.M., Batawi A.H. (2026). Targeting DNMT1 restores PTEN expression in colorectal cancer cells. Mol. Cell. Probes.

[35] Behera V., Evans P., Face C.J., Hamagami N., Sankaranarayanan L., Keller C.A., Giardine B., Tan K., Hardison R.C., Shi J. (2018). Exploiting genetic variation to uncover rules of transcription factor binding and chromatin accessibility. Nat. Commun..

[36] Zhang X.H., Jin X., Malladi S., Zou Y., Wen Y.H., Brogi E., Smid M., Foekens J.A., Massagué J. (2013). Selection of bone metastasis seeds by mesenchymal signals in the primary tumor stroma. Cell.

[37] Isozaki H., Sakhtemani R., Abbasi A., Nikpour N., Stanzione M., Oh S., Langenbucher A., Monroe S., Su W., Cabanos H.F. (2023). Therapy-induced APOBEC3A drives evolution of persistent cancer cells. Nature.

[38] Saranya I., Dharshini V.S., Akshaya R.L., Subhashini P.S., Selvamurugan N. (2024). Regulatory and therapeutic implications of competing endogenous RNA network in breast cancer progression and metastasis: A review. Int. J. Biol. Macromol..

[39] El-Ashmawy N.E., Khedr E.G., Abo-Saif M.A., Hamouda S.M. (2024). Long noncoding RNAs as regulators of epithelial mesenchymal transition in breast cancer: A recent review. Life Sci..

[40] Bao Q., Zhang H., Chen P., Wu S., Zou Y., Wang H., Cao J., Zhou W., Guan Z., Tang H. (2025). The Dual Roles of Circular RNAs in Breast Cancer Distant Metastasis and Their Clinical Applications. J. Cancer.

[41] Zhou W., Ye X.L., Xu J., Cao M.G., Fang Z.Y., Li L.Y., Guan G.H., Liu Q., Qian Y.H., Xie D. (2017). The lncRNA H19 mediates breast cancer cell plasticity during EMT and MET plasticity by differentially sponging miR-200b/c and let-7b. Sci. Signal..

[42] Zeng K., He B., Yang B.B., Xu T., Chen X., Xu M., Liu X., Sun H., Pan Y., Wang S. (2018). The pro-metastasis effect of circANKS1B in breast cancer. Mol. Cancer.

[43] Meng L., Liu S., Liu F., Sang M., Ju Y., Fan X., Gu L., Li Z., Geng C., Sang M. (2020). ZEB1-Mediated Transcriptional Upregulation of circWWC3 Promotes Breast Cancer Progression through Activating Ras Signaling Pathway. Mol. Ther. Nucleic Acids.

[44] Gregory P.A., Bert A.G., Paterson E.L., Barry S.C., Tsykin A., Farshid G., Vadas M.A., Khew-Goodall Y., Goodall G.J. (2008). The miR-200 family and miR-205 regulate epithelial to mesenchymal transition by targeting ZEB1 and SIP1. Nat. Cell Biol..

[45] Korpal M., Lee E.S., Hu G., Kang Y. (2008). The miR-200 family inhibits epithelial-mesenchymal transition and cancer cell migration by direct targeting of E-cadherin transcriptional repressors ZEB1 and ZEB2. J. Biol. Chem..

[46] Chaffer C.L., Marjanovic N.D., Lee T., Bell G., Kleer C.G., Reinhardt F., D’Alessio A.C., Young R.A., Weinberg R.A. (2013). Poised chromatin at the ZEB1 promoter enables breast cancer cell plasticity and enhances tumorigenicity. Cell.

[47] Dykxhoorn D.M., Wu Y., Xie H., Yu F., Lal A., Petrocca F., Martinvalet D., Song E., Lim B., Lieberman J. (2009). miR-200 enhances mouse breast cancer cell colonization to form distant metastases. PLoS ONE.

[48] Li X., Roslan S., Johnstone C.N., Wright J.A., Bracken C.P., Anderson M., Bert A.G., Selth L.A., Anderson R.L., Goodall G.J. (2014). MiR-200 can repress breast cancer metastasis through ZEB1-independent but moesin-dependent pathways. Oncogene.

[49] Ahmad A., Aboukameel A., Kong D., Wang Z., Sethi S., Chen W., Sarkar F.H., Raz A. (2011). Phosphoglucose isomerase/autocrine motility factor mediates epithelial-mesenchymal transition regulated by miR-200 in breast cancer cells. Cancer Res..

[50] Roy S.S., Gonugunta V.K., Bandyopadhyay A., Rao M.K., Goodall G.J., Sun L.Z., Tekmal R.R., Vadlamudi R.K. (2014). Significance of PELP1/HDAC2/miR-200 regulatory network in EMT and metastasis of breast cancer. Oncogene.

[51] El Nobey M.A., El-Hamidy S.M., Omar A.S., Al-Ghamdi K.M.S. (2026). 5-Azacytidine Reactivates E-cadherin via Promoter Demethylation and Induces Autophagy in Colorectal Cancer Cells. Egypt. J. Chem..

[52] El Nobey M.A., El-Hamidy S.M., Omar A.S., Ullah I., Hassan A. (2026). 5-aza-2-deoxycytidine Transiently Restores Dysregulated CDH1 Gene Expression in HNO-97 Cell Line. Egypt. J. Chem..

[53] Wee E.J., Peters K., Nair S.S., Hulf T., Stein S., Wagner S., Bailey P., Lee S.Y., Qu W.J., Brewster B. (2012). Mapping the regulatory sequences controlling 93 breast cancer-associated miRNA genes leads to the identification of two functional promoters of the Hsa-mir-200b cluster, methylation of which is associated with metastasis or hormone receptor status in advanced breast cancer. Oncogene.

[54] Le M.T., Hamar P., Guo C., Basar E., Perdigão-Henriques R., Balaj L., Lieberman J. (2014). miR-200-containing extracellular vesicles promote breast cancer cell metastasis. J. Clin. Investig..

[55] Ma L., Teruya-Feldstein J., Weinberg R.A. (2007). Tumour invasion and metastasis initiated by microRNA-10b in breast cancer. Nature.

[56] Dwedar F.I., Shams-Eldin R.S., Nayer Mohamed S., Mohammed A.F., Gomaa S.H. (2021). Potential value of circulatory microRNA10b gene expression and its target E-cadherin as a prognostic and metastatic prediction marker for breast cancer. J. Clin. Lab. Anal..

[57] Halim A., Kim B., Kenyon E., Moore A. (2025). miR-10b as a Clinical Marker and a Therapeutic Target for Metastatic Breast Cancer. Technol. Cancer Res. Treat..

[58] Ma L., Young J., Prabhala H., Pan E., Mestdagh P., Muth D., Teruya-Feldstein J., Reinhardt F., Onder T.T., Valastyan S. (2010). miR-9, a MYC/MYCN-activated microRNA, regulates E-cadherin and cancer metastasis. Nat. Cell Biol..

[59] Yang S., Li Y., Gao J., Zhang T., Li S., Luo A., Chen H., Ding F., Wang X., Liu Z. (2013). MicroRNA-34 suppresses breast cancer invasion and metastasis by directly targeting Fra-1. Oncogene.

[60] Li S.J., Wei X.H., Zhan X.M., He J.Y., Zeng Y.Q., Tian X.M., Yuan S.T., Sun L. (2020). Adipocyte-Derived Leptin Promotes PAI-1 -Mediated Breast Cancer Metastasis in a STAT3/miR-34a Dependent Manner. Cancers.

[61] Katzenellenbogen B.S., Guillen V.S., Katzenellenbogen J.A. (2023). Targeting the oncogenic transcription factor FOXM1 to improve outcomes in all subtypes of breast cancer. Breast Cancer Res..

[62] Tan X., Li Z., Ren S., Rezaei K., Pan Q., Goldstein A.T., Macri C.J., Cao D., Brem R.F., Fu S.W. (2019). Dynamically decreased miR-671-5p expression is associated with oncogenic transformation and radiochemoresistance in breast cancer. Breast Cancer Res..

[63] Krutilina R., Sun W., Sethuraman A., Brown M., Seagroves T.N., Pfeffer L.M., Ignatova T., Fan M. (2014). MicroRNA-18a inhibits hypoxia-inducible factor 1α activity and lung metastasis in basal breast cancers. Breast Cancer Res..

[64] Capaci V., Bascetta L., Fantuz M., Beznoussenko G.V., Sommaggio R., Cancila V., Bisso A., Campaner E., Mironov A.A., Wiśniewski J.R. (2020). Mutant p53 induces Golgi tubulo-vesiculation driving a prometastatic secretome. Nat. Commun..

[65] Yue X., Zhao Y., Zhang C., Li J., Liu Z., Liu J., Hu W. (2016). Leukemia inhibitory factor promotes EMT through STAT3-dependent miR-21 induction. Oncotarget.

[66] Douglass S., Meeson A.P., Overbeck-Zubrzycka D., Brain J.G., Bennett M.R., Lamb C.A., Lennard T.W., Browell D., Ali S., Kirby J.A. (2014). Breast cancer metastasis: Demonstration that FOXP3 regulates CXCR4 expression and the response to CXCL12. J. Pathol..

[67] Zhang G., Zhang W., Li B., Stringer-Reasor E., Chu C., Sun L., Bae S., Chen D., Wei S., Jiao K. (2017). MicroRNA-200c and microRNA- 141 are regulated by a FOXP3-KAT2B axis and associated with tumor metastasis in breast cancer. Breast Cancer Res..

[68] El-Khazragy N., Alsolami A., Aref A.M., Hassan M.N.M., Othman M.S. (2026). MiR-200c restoration inhibits FOXP3 and metastatic spread in breast cancer: Evidence from in vitro and in vivo models. BMC Cancer.

[69] Woo Y.L., Sterling J., Crawford R., van der Burg S.H., Coleman N., Stanley M. (2008). FOXP3 immunohistochemistry on formalin-fixed paraffin-embedded tissue: Poor correlation between different antibodies. J. Clin. Pathol..

[70] Takenaka M., Seki N., Toh U., Hattori S., Kawahara A., Yamaguchi T., Koura K., Takahashi R., Otsuka H., Takahashi H. (2013). FOXP3 expression in tumor cells and tumor-infiltrating lymphocytes is associated with breast cancer prognosis. Mol. Clin. Oncol..

[71] Sadaf, Akhter N., Alharbi R.A., Sindi A.A.A., Najm M.Z., Alhumaydhi F.A., Khan M.A., Deo S.V.S., Husain S.A. (2021). Epigenetic Alteration and its Association With Downregulated FOXP3 Gene in Indian Breast Cancer Patients. Front. Genet..

[72] Damiano V., Brisotto G., Borgna S., di Gennaro A., Armellin M., Perin T., Guardascione M., Maestro R., Santarosa M. (2017). Epigenetic silencing of miR-200c in breast cancer is associated with aggressiveness and is modulated by ZEB1. Genes Chromosomes Cancer.

[73] Yan W., Cao Q.J., Arenas R.B., Bentley B., Shao R. (2010). GATA3 inhibits breast cancer metastasis through the reversal of epithelial-mesenchymal transition. J. Biol. Chem..

[74] Tavazoie S.F., Alarcón C., Oskarsson T., Padua D., Wang Q., Bos P.D., Gerald W.L., Massagué J. (2008). Endogenous human microRNAs that suppress breast cancer metastasis. Nature.

[75] Zhang L., Sullivan P.S., Goodman J.C., Gunaratne P.H., Marchetti D. (2011). MicroRNA-1258 suppresses breast cancer brain metastasis by targeting heparanase. Cancer Res..

[76] Li J.T., Jia L.T., Liu N.N., Zhu X.S., Liu Q.Q., Wang X.L., Yu F., Liu Y.L., Yang A.G., Gao C.F. (2015). MiRNA-101 inhibits breast cancer growth and metastasis by targeting CX chemokine receptor 7. Oncotarget.

[77] Chen W.J., Ye Q.Q., Wu H.T., Wu Z., Lan Y.Z., Fang Z.X., Lin W.T., Liu J. (2024). MiR-338-5p, a novel metastasis-related miRNA, inhibits triple-negative breast cancer progression by targeting the ETS1/NOTCH1 axis. Heliyon.

[78] Zhou W., Fong M.Y., Min Y., Somlo G., Liu L., Palomares M.R., Yu Y., Chow A., O’Connor S.T., Chin A.R. (2014). Cancer-secreted miR-105 destroys vascular endothelial barriers to promote metastasis. Cancer Cell.

[79] Fong M.Y., Zhou W., Liu L., Alontaga A.Y., Chandra M., Ashby J., Chow A., O’Connor S.T., Li S., Chin A.R. (2015). Breast-cancer-secreted miR-122 reprograms glucose metabolism in premetastatic niche to promote metastasis. Nat. Cell Biol..

[80] Tominaga N., Kosaka N., Ono M., Katsuda T., Yoshioka Y., Tamura K., Lötvall J., Nakagama H., Ochiya T. (2015). Brain metastatic cancer cells release microRNA-181c-containing extracellular vesicles capable of destructing blood-brain barrier. Nat. Commun..

[81] Othman M.S., Elabbasy M.T., Aref A.M., Altaleb A.A., Mohammed M.H., Soliman D.A.M., El-Khazragy N. (2024). The MiR-200c/FOXP3 Network: A Promising Biomarker for Predicting Trastuzumab Response in HER2-Positive Breast Cancer. Technol. Cancer Res. Treat..

[82] Pritchard C.C., Kroh E., Wood B., Arroyo J.D., Dougherty K.J., Miyaji M.M., Tait J.F., Tewari M. (2012). Blood cell origin of circulating microRNAs: A cautionary note for cancer biomarker studies. Cancer Prev. Res..

[83] Arroyo J.D., Chevillet J.R., Kroh E.M., Ruf I.K., Pritchard C.C., Gibson D.F., Mitchell P.S., Bennett C.F., Pogosova-Agadjanyan E.L., Stirewalt D.L. (2011). Argonaute2 complexes carry a population of circulating microRNAs independent of vesicles in human plasma. Proc. Natl. Acad. Sci. USA.

[84] Welsh J.A., Goberdhan D.C.I., O’Driscoll L., Buzas E.I., Blenkiron C., Bussolati B., Cai H., Di Vizio D., Driedonks T.A.P., Erdbrügger U. (2024). Minimal information for studies of extracellular vesicles (MISEV2023): From basic to advanced approaches. J. Extracell. Vesicles.

[85] Wang K., Yuan Y., Cho J.H., McClarty S., Baxter D., Galas D.J. (2012). Comparing the MicroRNA spectrum between serum and plasma. PLoS ONE.

[86] Kirschner M.B., Kao S.C., Edelman J.J., Armstrong N.J., Vallely M.P., van Zandwijk N., Reid G. (2011). Haemolysis during sample preparation alters microRNA content of plasma. PLoS ONE.

[87] Elango R., Alsaleh K.A., Vishnubalaji R., Manikandan M., Ali A.M., Abd El-Aziz N., Altheyab A., Al-Rikabi A., Alfayez M., Aldahmash A. (2020). MicroRNA Expression Profiling on Paired Primary and Lymph Node Metastatic Breast Cancer Revealed Distinct microRNA Profile Associated With LNM. Front. Oncol..

[88] Mitchell P.S., Parkin R.K., Kroh E.M., Fritz B.R., Wyman S.K., Pogosova-Agadjanyan E.L., Peterson A., Noteboom J., O’Briant K.C., Allen A. (2008). Circulating microRNAs as stable blood-based markers for cancer detection. Proc. Natl. Acad. Sci. USA.

[89] Antolín S., Calvo L., Blanco-Calvo M., Santiago M.P., Lorenzo-Patiño M.J., Haz-Conde M., Santamarina I., Figueroa A., Antón-Aparicio L.M., Valladares-Ayerbes M. (2015). Circulating miR-200c and miR-141 and outcomes in patients with breast cancer. BMC Cancer.

[90] Madhavan D., Peng C., Wallwiener M., Zucknick M., Nees J., Schott S., Rudolph A., Riethdorf S., Trumpp A., Pantel K. (2016). Circulating miRNAs with prognostic value in metastatic breast cancer and for early detection of metastasis. Carcinogenesis.

[91] Fischer C., Turchinovich A., Feisst M., Riedel F., Haßdenteufel K., Scharli P., Hartkopf A.D., Brucker S.Y., Michel L., Burwinkel B. (2022). Circulating miR-200 Family and CTCs in Metastatic Breast Cancer before, during, and after a New Line of Systemic Treatment. Int. J. Mol. Sci..

[92] Pixberg C.F., Raba K., Müller F., Behrens B., Honisch E., Niederacher D., Neubauer H., Fehm T., Goering W., Schulz W.A. (2017). Analysis of DNA methylation in single circulating tumor cells. Oncogene.

[93] Altman D.G., McShane L.M., Sauerbrei W., Taube S.E. (2012). Reporting recommendations for tumor marker prognostic studies (REMARK): Explanation and elaboration. BMC Med..

[94] Sturzu A., Ma R., Xi Y. (2026). Circulating MicroRNA in Breast Cancer. Cancers.

[95] Podder P.S., Bhadra D., Martinelli C., Cortellino S., Giordano A., Cinti S. (2026). Liquid biopsy in breast cancer: Current biomarker platforms and clinical applications. Explor. Target. Antitumor Ther..

[96] Lou Y., Wang Y., Lu J., Chen X. (2025). MicroRNA-targeted nanoparticle delivery systems for cancer therapy: Current status and future prospects. Nanomedicine.

[97] Song D., Zhao Y., Wang Z., Xu Q. (2024). Tuning Lipid Nanoparticles for RNA Delivery to Extrahepatic Organs. Adv. Mater..

[98] Hong D.S., Kang Y.K., Borad M., Sachdev J., Ejadi S., Lim H.Y., Brenner A.J., Park K., Lee J.L., Kim T.Y. (2020). Phase 1 study of MRX34, a liposomal miR-34a mimic, in patients with advanced solid tumours. Br. J. Cancer.

[99] Takahashi R.U., Miyazaki H., Takeshita F., Yamamoto Y., Minoura K., Ono M., Kodaira M., Tamura K., Mori M., Ochiya T. (2015). Loss of microRNA-27b contributes to breast cancer stem cell generation by activating ENPP1. Nat. Commun..

